# Hypoglycemia revealing arachnoidocele in infant

**DOI:** 10.11604/pamj.2015.22.46.7814

**Published:** 2015-09-18

**Authors:** Manel Jellouli, Tahar Gargah

**Affiliations:** 1 Department of Pediatric, Charles Nicolle Hospital, Tunis, Tunisia

**Keywords:** Hypoglycemia, arachnoidocele, herniation, subarachnoid space

## Image in medicine

Arachnoidocele is characterized by the herniation of the subarachnoid space within the sella turcica, associated with some degree of flattening of the pituitary gland. In children and adolescents, Arachnoidocele is rare, and the clinical picture is much less benign, with an increase in familial incidence, associated skeletal disorders, and endocrine abnormalities. OH was in infant admitted at the age of one month for respiratory symptoms. The examination on admission found an hypothermic infant with macroglossia, an anterior and posterior fontanelle wide, an umbilical hernia with growth retardation and normal cranial perimeter. Her height was 3200 g and her weight was 52 cm. The examination of others systems was unremarkable. She had central hypothyroidism (FT4 below 0.4 and TSH equal to 0.346). An exploration of the pituitary was requested, finding an achievement of the cortical axis revealed by the occurrence of multiple episodes of hypoglycemia with normal insulinemia and normal Adreno-Cortico-Trophic-Hormone with low cortisolemia (52 nmol/l). Growth stimulation tests were performed. Growth hormone levels were less than 10 ng/ml. A brain MRI was applied for a arachnoidocele intra-sellar repressing anterior pituitary with an anterior pituitary parenchyma very small without abnormality posterior pituitary or pituitary stalk. The patient was treated with hydrocortisone, levothyroxine and growth hormone replacement. The outcome was favorable without recourse to neurosurgery at the lack of damage to the optic nerve and the sphenoid bone, with a decline of five years otherwise the child has a delay in psychomotor acquisitions.

**Figure 1:**
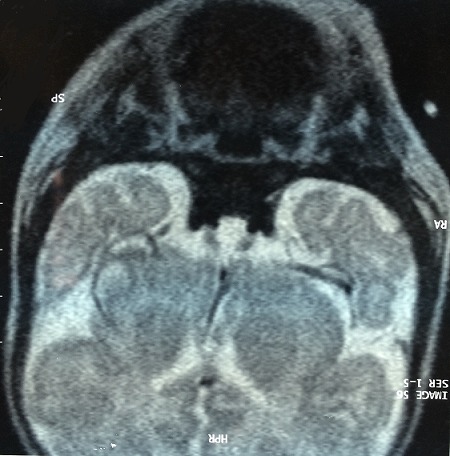
MRI of total empty sella: Coronal weighted T1 image showing arachnoidocele intra-sellar

